# Supplementary material for: Efficacy of different courses of acupuncture for diarrhea irritable bowel syndrome: A protocol for systematic review and meta-analysis
Source: PLoS One. 2023 Dec 14;18(12):e0295077. doi: 10.1371/journal.pone.0295077 (PMC10721030; doi:10.1371/journal.pone.0295077)
Supplement: Attachment — Submitted filename: Response to Reviewers.doc [file pone.0295077.s002.doc]

Dear editors:

On behalf of our co-authors, we thank you very much for giving us an opportunity to revise our manuscript, we highly appreciate reviewers for their positive and constructive comments and suggestions on our manuscript entitled “Efficacy of Different Courses of Acupuncture for Diarrhea Irritable bowel syndrome: a protocol for systematic review and meta-analysis” (PONE-D-23-13895). We have revised the manuscript which is marked in red or bule in the paper, according to the comments and suggestions of reviewers, and responded, point by point to, the comments as listed below:

Response to Editorial Team:

1.Thoroughly copyedit your manuscript for language usage, spelling, and grammar.

Response: Dear editor, We appreciate the Editor’s comment.

We've carefully checked the language usage, spelling, and grammar of the manuscript.

2.Provide line numbers in your PRISMA-P checklist, specifying the location of the relevant information in the manuscript,

Response: Dear editor, We appreciate the Editor’s comment.

We have provided the line numbers for the relevant information in my PRISMA-P checklist

Response to reviewer:

Have the authors described where all data underlying the findings will be made available when the study is complete?

The [PLOS Data policy](https://journals.plos.org/plosone/s/materials-and-software-sharing) requires authors to make all data underlying the findings described in their manuscript fully available without restriction, with rare exception, at the time of publication. The data should be provided as part of the manuscript or its supporting information, or deposited to a public repository. For example, in addition to summary statistics, the data points behind means, medians and variance measures should be available. If there are restrictions on publicly sharing data—e.g. participant privacy or use of data from a third party—those must be specified.

Reviewer #1: Yes

Reviewer #2: Yes

Reviewer #3: No

Response: Dear reviewer, thank you for your comments.

We appreciate the reviewer’s comment. In the submission system, I have described that the relevant data will be made public after the study is completed.

No datasets were generated or analysed during the current study. All relevant data from this study will be made available upon study completion.”

Reviewer #1: The protocol is well written and meets all the methodological requirements for the conduct an adequate systematic review and meta-analysis.

I have only two considerations to make:

1. It is essential for the authors to add information in the data synthesis section about how the results will be presented if conducting the meta-analysis is not possible.

Response: Dear reviewer, We agree with the reviewer’s comment.

Thank you very much for your advice! I will be adding relevant information to the article.We will present a narrative synthesis when heterogeneity is too apparent to resolve.

2. I suggest the authors use the Rayyan tool for the exclusion of duplicates; and selection of studies. Free tool designed exclusively for conducting systematic reviews.

Response: Dear reviewer, We agree with the reviewer’s comment.

EedNote software has the function of automatically finding duplicates, and we will also carry out manual exclusion of duplicates to ensure the quality of the literature.

Reviewer #2: The authors present a systematic review protocol whose objective is to evaluate effectiveness of Acupuncture of different courses in the treatment of IBS-D and provide sufficient evidence for clinical recommendations for IBS-D. The manuscript was very well written and contemplates what is recommended in PRISMA-P.
Only , I suggest clarification on which validated tools should have been used in the RCTs to assess the primary and secondary outcomes defined by the authors.
"Efficiency rate and the Symptoms Severity Score", which are the primary ones, probably have validated instruments and I suggest that you include the main ones with references. Likewise, there are validated instruments for abdominal pain scores, abdominal frequency scores, satisfaction with bowel habits scores and quality of life and the authors must mention which ones will be considered and cite their references in the manuscript.

Response: Dear reviewer, We acknowledge the reviewer’s comment.

Thank you for your advice! I will add the relevant content and apply the validated tools to define validity. The details are as follows:

The primary outcome measures of this review will be the efficiency rate and the Symptoms Severity Score [1]. The clinical efficiency rate was defined as the degree of relief of IBS symptoms, which are assessed through the Symptoms Severity Score. This includes the use of the IBS Symptom Severity Scale (IBS-SSS)[2], or the Gastrointestinal Symptom Rating Scale for IBS (GSRS-IBS)[3].

Reviewer #3: In general, the paper is well-written and logically presented. However, there are a few points that require your attention：
1.Inclusion criteria

1.1 Diagnostic criteria for IBS-D lack references.

Response: Dear reviewer, We appreciate the reviewer’s comment.

We will add references to the diagnostic criteria for IBS-D.The patients diagnosed with IBS-D will be eligible based on Rome IV/III/II criteria[4-7].

1.2 Scoring criteria for outcome indicators were not explained.

Response: Dear reviewer, We appreciate the reviewer’s comment.

We will add the relevant content and apply the validated tools to define validity. The details are as follows:

The primary outcome measures of this review will be the efficiency rate and the Symptoms Severity Score [1]. The clinical efficiency rate was defined as the degree of relief of IBS symptoms, which are assessed through the Symptoms Severity Score. This includes the use of the IBS Symptom Severity Scale (IBS-SSS)[2], or the Gastrointestinal Symptom Rating Scale for IBS (GSRS-IBS)[3].

2.Many formatting errors in this article.

Response: Dear reviewer, We appreciate the reviewer’s comment.

I'm going to carefully revise the formatting of my post with a view to achieving good results.

3. The title is "Efficacy of Different Courses of Acupuncture for Diarrhea Irritable bowel syndrome: a protocol for systematic review and meta-analysis", but the inclusion criteria is "acupuncture or EA", please explain.

Response: Dear reviewer, We appreciate the reviewer’s comment.

Acupuncture, including manual acupuncture (also known as Acupuncture) and electroacupuncture, is a commonly used therapeutic technique in clinical practice. Most meta-analysis studies on acupuncture have included electroacupuncture as an intervention.

We would like to resubmit this revised manuscript to “PLOS ONE” and hope that it will be acceptable for publication in the journal.

We look forward to hearing from you soon.

References

1.	Wang M, Xie X, Zhao S, Ma X, Wang Z, Zhang Y. Fecal microbiota transplantation for irritable bowel syndrome: a systematic review and meta-analysis of randomized controlled trials. Frontiers in immunology. 2023;14:1136343.

2.	Francis CY, Morris J, Whorwell PJ. The irritable bowel severity scoring system: a simple method of monitoring irritable bowel syndrome and its progress. Alimentary pharmacology & therapeutics. 1997;11(2):395-402.

3.	Wiklund IK, Fullerton S, Hawkey CJ, Jones RH, Longstreth GF, Mayer EA, et al. An irritable bowel syndrome-specific symptom questionnaire: development and validation. Scandinavian journal of gastroenterology. 2003;38(9):947-54.

4.	Badia X, Mearin F, Balboa A, Baro E, Caldwell E, Cucala M, et al. Burden of illness in irritable bowel syndrome comparing Rome I and Rome II criteria. PharmacoEconomics. 2002;20(11):749-58.

5.	Markert C, Suarez-Hitz K, Ehlert U, Nater UM. Endocrine dysregulation in women with irritable bowel syndrome according to Rome II criteria. J Behav Med. 2016;39(3):519-26.

6.	Drossman DA, Dumitrascu DL. Rome III: New standard for functional gastrointestinal disorders. Journal of gastrointestinal and liver diseases : JGLD. 2006;15(3):237-41.

7.	Barberio B, Houghton LA, Yiannakou Y, Savarino EV, Black CJ, Ford AC. Symptom Stability in Rome IV vs Rome III Irritable Bowel Syndrome. The American journal of gastroenterology. 2021;116(2):362-71.
